# Detection of *Enterococcus hirae* in a case of acute osteomyelitis

**DOI:** 10.1016/j.radcr.2021.06.016

**Published:** 2021-07-01

**Authors:** Rahul Bollam, Mohamed Yassin, Tung Phan

**Affiliations:** a Department of Medicine, University of Pittsburgh Medical Center, Pittsburgh, Pennsylvania, USA; b Division of Clinical Microbiology, University of Pittsburgh and University of Pittsburgh Medical Center, Pittsburgh, Pennsylvania, USA

**Keywords:** Enterococcus *hirae*, osteomyelitis, trauma, fracture, VITEK 2

## Abstract

Enterococci are important microorganisms of the gut microbiome in many mammals and cause millions of infections annually. An increasing resistance to antibiotics has led to their emergence as superinfecting nosocomial pathogens in humans. *Enterococcus hirae* is rarely identified in humans. In this study, we present a case of the polymicrobial osteomyelitis involving *Enterococcus hirae* in a 33-year-old male patient with traumatic tibia-fibula fracture after a motor vehicle accident. He underwent a right below-the-knee amputation and antibiotics with excellent improvement. Our case study helps to confirm the unexpected presence of *Enterococcus hirae* in a human specimen. Further studies are needed to elucidate the clinical implications of *Enterococcus hirae*.

## Introduction

Enterococci are ubiquitous microorganisms, and they are among the most common nosocomial pathogens in humans [1]. Enterococci could cause a wide variety of important infections such as bacteremia, endocarditis, urinary tract infection, intra-abdominal abscesses, and central nervous system infection. While *E. faecalis* and *E. faecium* are the most commonly human pathogens, *E. hirae* is well documented to cause infections in animals. *E. hirae* was reported to be associated with diarrhea in rats, endocarditis in chickens, mastitis in cattle, and ascending cholangitis & ductal pancreatitis in cats [2]. Septicemia due to *E. hirae* was identified in ten different species of psittacine birds [3]. *E. hirae* was also detected frequently from feces of healthy psittacine birds, suggesting that this bacterium is a member of the normal intestinal flora of these birds [3]. In this study, we present the first case of an isolated *E. hirae* in wound culture, which was identified in a patient who had traumatic tibia-fibula fracture after open reduction and fixation.

## Case presentation

**Fig. 1:**
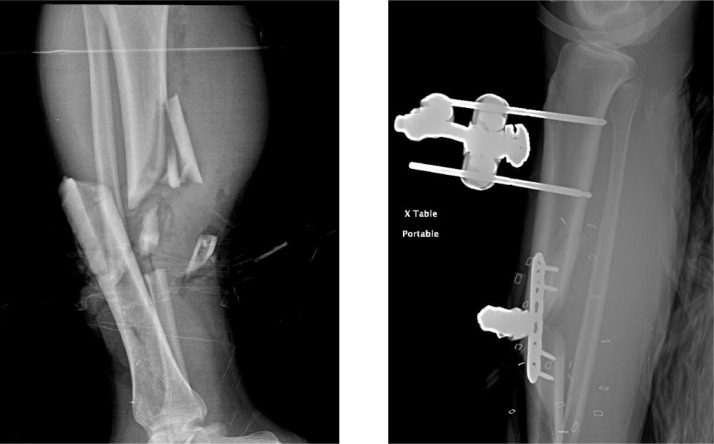
The radiograph of the right tibia-fibula fracture before (*left*) and after (*right*) the open reduction internal fixation (ORIF) and external fixation device application.

A 33-year-old male with no significant past medical history presented to the Emergency Department after a motor vehicle accident with a 10 cm laceration to his proximal left arm, an open right tibial fracture with deformity, and traumatic disruption of his arterial blood supply to the right lower extremity. At the time of admission, the patient was afebrile (37.4°C), and had blood pressure of 107/55 mmHg, heart rate 99 bpm, respiratory rate 17/min, and blood oxygen saturation level 100%. He had equal air entry in both lung fields with normal breath sounds. No lymphadenopathy was found. His oral mucosa showed no signs of pathology. There were no focal neurological deficits. The patient had underwent multiple surgeries and procedures including the open reduction internal fixation (ORIF) of the right tibia, external fixation device application (Fig. 1), latissimus dorsi free flap reconstruction, skin grafting, repair of right posterior tibial artery, incision & drainage, and wound VAC placements throughout his hospital stay. However, the flap did not heal well, and acute osteomyelitis was developed due to *Stenotrophomonas* sp. and *E. hirae* after 2 weeks of admission. There was no evidence of sepsis during the clinical course. The blood culture was also performed, but it was negative. The patient also lost the sensation & motor function of right foot extending up to the middle of the lower leg. He underwent a right below-the-knee amputation and had an uncomplicated postoperative course. The patient was subsequently placed on six weeks of antibiotics (vancomycin and levofloxacin), with excellent improvement.

## Discussion

**Fig. 2:**
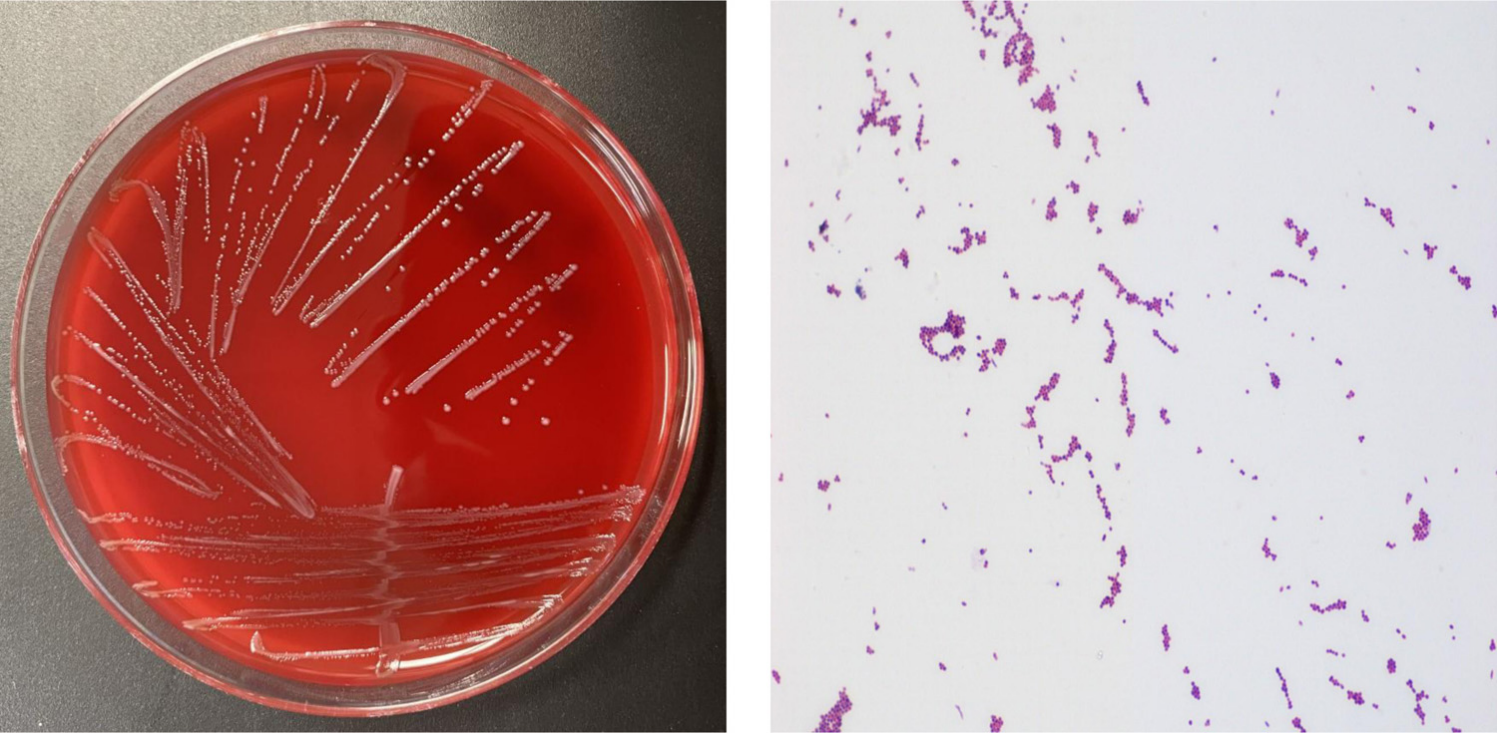
Small, gray, non-hemolytic colonies of *Enterococcus hirae* were observed on sheep blood agar, and microscopic examination of a Gram-stained smear revealed small Gram-positive cocci at 1000 x magnification.

A bone specimen through incision and drainage was submitted to our laboratory for bacterial and fungal cultures. After 48 hours of incubation at 35°C in 5% CO2, growth was observed on nonselective blood and chocolate agar plates. Small, gray, non-hemolytic colonies grew on selective Columbia CNA agar plate. Microscopic examination of a gram-stained smear revealed gram-positive cocci (Fig. 2). The identification was performed by the VITEK 2 system, and the isolate was identified as *E. hirae* with an excellent confidence score of 99%. *E. hirae* was found to be sensitive to drugs (ampicillin, gentamicin and vancomycin) tested in the laboratory. Other clear, lactose-negative colonies were also seen to grow on selective MacConkey agar plate. These colonies were gram-negative bacilli, being identified as *Stenotrophomonas* sp. that has been known to be associated with osteomyelitis of tibia, skull base, vertebrae, and pelvis [4], [5], [6], [7], [8]. *Stenotrophomonas* sp. was found in a case of polymicrobial osteomyelitis in which *Streptococcus anginosus* and *Granulicatella adiacens* were also isolated [9]. Hagiya et al. reported the recurrent *Stenotrophomonas* bacteremia due to osteomyelitis caused by *Stenotrophomonas* sp. following an iliac crest bone graft harvest [10].

*Enterococci* are gram-positive, catalase-negative, nonspore-forming bacteria. There are approximately 58 different species belonging to the genus *Enterococcus. E. hirae* was first named in 1985 when this bacterium was found to cause growth depression in young chickens [11]. Since then, *E. hirae* has been often found in different animals, but extremely rare cases were described in humans. The first human infection caused by *E. hirae* was discovered in blood collected from a patient with end-stage renal disease undergoing hemodialysis in 1998 [12]. Winther and co-authors found the first case of infective endocarditis caused by *E. hirae* in Denmark, which was the sixth case worldwide [13]. This bacterium has been also reported to cause urinary tract infections, pneumonia, spondylodiscitis, pancreatitis in only a small number of adult cases [13]. In 2019, the first pediatric case of *E. hirae* bacteremia was documented in a 7-month-old boy who was born prematurely in Puerto Rico at 33 weeks’ gestation [14]. However, there is the low incidence of *E. hirae* infections in humans. A study from Saudi Arabia reported the very low detection rate of *E. hirae* when 206 clinical isolates of enterococci were identified to species level [15]. While *E. hirae* was found in only 0.8%, *E. faecalis* was predominant (69.2%), *E. faecium* (11.3%), *E. avium* (2.1%), *E. casseliflavus* (1.3%) and *E. gallinarum* (1.3%) [15]. In addition, *E. hirae* may be underdiagnosed or misdiagnosed by some bacterial identification methods such as API 20 Strep, and Rapid ID 32 STREP [16]. In the study, we presented a unique case of acute osteomyelitis involving *E. hirae* in an adult patient with traumatic tibia-fibula fracture after repair. It is unclear whether *E. hirae* plays any role in causing acute osteomyelitis in this clinical case since *Stenotrophomonas* sp. was also isolated from the site. Further studies are needed to investigate the pathogenicity of *E. hirae*. It is found that *E. hirae* infections are increasingly reported, so we should consider this microorganism as a clinical pathogen. Since the prevalence and dissemination of multidrug-resistant *Enterococcus* sp. worldwide have increased, the antimicrobial profile of *E. hirae* should be monitored.

## Ethical approval

Approval from the ethical committee was not required due to the nature of this case report. Abiding by the Declaration of Helsinki, patient anonymity was guaranteed.

## Author contributions

TP, RB and MY: designed the study and wrote the manuscript.
